# Biochemical and histologic changes in rats after prolonged administration of the crude aqueous extract of the leaves of *Vitex grandifolia*

**DOI:** 10.4103/0974-8490.72322

**Published:** 2010

**Authors:** Mbang A. Owolabi, Moyosola M. Abass, Promise M. Emeka, Smith I. Jaja, Martin Nnoli, Benjamin O. S. Dosa

**Affiliations:** *Department of Pharmaceutical Chemistry, Natural Product Laboratory, Faculty of Pharmacy, College of Medicine, University of Lagos, Lagos, Nigeria*; 1 *Department of Pharmacology, College of Medicine, University of Lagos. P.M.B. 12003, Lagos, Nigeria*; 2 *Department of Physiology, College of Medicine, University of Lagos. P.M.B. 12003, Lagos, Nigeria*; 3 *Department of Morbid Anatomy, College of Medicine, University of Lagos. P.M.B. 12003, Lagos, Nigeria*

**Keywords:** Histopathology, liver function test, edema, portal infi ltration, toxicity, *Vitex grandifolia*

## Abstract

**Background:**

In recent times, many herbal remedies are used to treat variety of ailments. The leaves of *Vitex grandifolia* is claimed to be effective in the treatment of diabetes mellitus and as a diuretic in the treatment of high blood pressure. However, there are no scientific reports on the therapeutic benefits or toxicity of this plant. This study therefore investigated the effect of prolonged administration of the aqueous extract of the leaves of this plant in Sprague–Dawley rats.

**Methods:**

The plant leaves (No. FHI 107055) were dried at 40° C, powdered and extracted at room temperature in water (pH 5.72) by percolation. Extract was dried in *vacuo* to give a yield of 27.32 %w/v. The extract, 0.5–2 g/kg b. wt. was administered by gastric probe to rats for 14 days. The liver and kidney functions, blood chemistry, histopathologic alterations of vital organs and extract effect on rats b. wt. were investigated.

**Results:**

*V. grandifolia* caused significant increase in the serum electrolytes, creatinine, and liver function enzyme dose dependently compared with the control (*P*≤ 0.001). The extract had no effect on the heart; however, the architecture of the liver, kidney, and lungs were significantly altered in the treated groups compared with the control. The treated rats had significantly reduced body weight compared with the control (*P*≤ 0.001). Major clinical signs observed in the treated groups were polydipsia, polyuria, puffiness of hair, and calmness, which were consistent with increase in dose of the extract.

**Conclusion:**

It could be clearly concluded that prolonged administration of the aqueous leaf extract of *V. grandifolia* at the dose used in this study tends to be toxic to the rats. Its use in folkloric medicine should be with utmost care.

## INTRODUCTION

Since the dawn of history, nature and natural sources, such as plants, animals, microbes, and minerals have remained a veritable source of bioactive compounds with medicinal values. Plants have been most explored and exploited for their bioactive medicinal components, which have always served as “lead compounds” or templates for the rational development of drugs with more specific efficacies and less side effects for human use.

Many herbal remedies are frequently used to treat a variety of ailments and symptoms with little or no information about their efficacy or toxicity. *Vitex grandifolia* Gürke (Verbenaceae), an annual plant with stem creeping and sometimes ascending is found in some West African countries extending from Sierra Leone east to Cameroon and Gabon.[1] The plant measures about 10–12 cm in length and 5–7 cm in width. It is known in local names as Oori ọdan (Yoruba, Nigeria), ofonma (Egun, Republic of Benin), or ofofrin (Setangun, Republic of Benin). The fruits of this plant are edible and used to make an alcoholic drink, the bark is used as a stomachic and to treat diarrhea, bronchial complaints, rickets, sore, and fever. The leaves are used in medication against colic, infections of the umbilical cord, toothache, rheumatism, and orchitis.[1] The powder leaf of *V. grandifolia* has been reported to have a biocidal effect against *Tribolium castaneum* in stored groundnut *Arachis hypogaea*.[2]

Among the Egun and Setangun tribes of the Republics of Nigeria and Benin, an excellent use is claimed of the infusion of the aqueous extract of the leaves of this plant for the treatment of diabetes mellitus and as a diuretic in the treatment of high blood pressure (Dosa, 2007, personal communication). However, there are no scientific reports on the therapeutic benefits or toxicity of the leaf extract of *V. grandifolia*. This study would therefore evaluate the effect of prolonged administration of the aqueous extract of the leaves of this plant on blood chemistry, liver functions, and histopathology of some vital organs in an effort to establish its toxicological implications.

## MATERIALS AND METHODS

### Plant material and preparation of extract

*V. grandifolia* leaves, collected from Egun in Ipokia Local Government of Ogun State, Nigeria, in March 2007, were authenticated by Mr. T. K. Odewo, Forestry Research Institute of Nigeria (FRIN), Ibadan. A voucher specimen has been deposited in the FRIN herbarium (No. FHI 107055). The plant material was dried at room temperature in the laboratory for 5 days. The powdered plant material, 500 g was extracted at room temperature in water (pH 5.72) by percolation. The crude water extract was filtered and concentrated to dryness at 40°C to give 136.62 g representing a percentage yield of 27.32 %w/v. The extract residue was stored in an impervious airtight amber bottle until use.

### Chemical and instrument

All reagents were of analytical grade purchased from Sigma Chemical Co., London. The UV–Vis spectrophotometer was Agilent 8453 (Serial No. CN02500898; Product No. G1103A; Agilent Technologies, Hewlett-Packard, Germany). The absorbances were recorded by Agilent ChemStation software (Agilent, Palo Alto, USA) running on a Compaq compatible personal computer (Hewlett-Packard, Obregon, Mexico) with an Intel Pentium processor operating at 2799 MHz under the Microsoft (Redmond, WA) Windows operating environment. The ChemStation also consists of an interface bus for data acquisition and a pinwriter HP DeskJet 5652 printer. The Sychron CX5 automated system was Beckman instrument Inc. Diagnostic Systems Group, 200 S. Kraemer Blvd. Brea, California 92621-6209, USA. (PTS Model: 7579; Catalog No. 757950; Serial No. 4562).

### Animals

Sprague–Dawley albino rats of either sex weighing between 220 and 230 g were used for the studies. The animals were cared for and used in accordance with the Institute of Laboratory Animal Research (ILAR) guidelines for care and use of animals in experimental studies.[3] The animals were obtained from the Laboratory Animal Care Center of the College of Medicine, University of Lagos, Lagos, Nigeria, and were kept in a well-ventilated animal house. The animals that were acclimatized for 14 days before commencement of the study received standard rat chow (Pfizer Feeds Plc., Ibadan, Nigeria) and water ad libitum.

### Toxicity studies

The acute toxicity was studied according to the method of Organization for Economic Co-operation and Development (OECD) guidelines.[4] Forty rats divided into 4 equal groups were fasted for 24 h prior to the study. Doses of the extract, 0.5, 1.0, and 2.0 g/kg body weight, were administered orally by gastric probe; the control group received equivalent volume of the injection vehicle (distilled water). Mortality was determined 24 h postadministration and the LD_50_ calculated. The animals were observed for behavioral changes and a further 14 days observation period was allowed on test animals for the elimination of delayed toxicity symptoms, which usually occur in some drugs. In another experiment, 4 groups of 10 rats each were used. Groups 1–3 were administered intragastrally, 0.5, 1.0, and 2.0 g/kg body weight extract daily for 14 days, respectively, with repeated weighing. The 4th group received distilled water for the same number of days. The animals were allowed access to food and water ad libitum. After 24 h of extract administration, 5 mL of blood sample was withdrawn from the heart of each rat into lithium heparinized bottles under mild chloroform anesthesia. Each blood sample was centrifuged at 10,000 rpm for 10 min and the plasma, which was used for the biochemical studies was promptly freezed at –20°C until assayed.

### Histopathologic studies

Histopathologic examination was carried out on the liver, heart, lungs, and kidney of the control and test groups. At postdose, the animals were sacrificed by cervical dislocation under mild chloroform anesthesia and the abdomen were cut open to remove the liver, kidney, lungs, and heart. The organs were fixed with 10% formalin solution for 12 h and embedded in paraffin.[5] Five micron (5 µm) cryostat sections were stained with hematoxylin and eosin dyes and periodic acid Schiff reagent without and with diastase, respectively. The sections were examined under light microscope at high power magnifications for changes in organ architecture and photomicrographs were taken.

### Biochemical studies

#### Determination of plasma creatinine

The plasma creatinine was estimated using Jaffe reaction as described by Perrone *et al*., 1992.[6] In this reaction, creatinine reacts with picrate ion in an alkaline medium to yield an orange-red complex. Briefly, 1 mL plasma was diluted with 3 mL distilled water and mixed thoroughly followed by the addition of 2 mL of 5% sodium tungstate solution. Two milliliters of 0.67 N sulfuric acid was added dropwise to the mixture, shaken vigorously and allowed to stand for 20 min, then centrifuged at 3000 rpm for 15 min. To 3 mL of the supernatant was added 1 mL saturated picric acid solution and 1 mL, 0.75 N NaOH, mixed and allowed to stand for 15 min. The absorbance of the solution was read at 525 nm against a reagent blank. A calibration curve of creatinine was prepared and the plasma creatinine was obtained from the calibration function.

#### Determination of plasma aspartate and alanine transaminases

The transaminases were estimated by the method of Karmen *et al*., 1955.[7] The assay principle is based on the fact that aspartate (AST) or alanine (ALT) transaminases catalyse the transfer of the amino group from aspartate or alanine, respectively, to oxoglutarate, which is subsequently converted to pyruvate. The pyruvate then reacts with a chromogen to give a brown colored hydrazone, which is measured spectrophotometrically. In brief, 0.1 mL plasma was added to 0.5 mL buffered substrate, pH 7.5 in different tubes and incubated in a water bath at 37°C for 60 min (AST) or 30 min (ALT). Two milliliters of the chromogen, 2,4-dinitrophenylhydrazine reagent was added to each tube, mixed, and allowed to stand for 20 min. Five milliliters of 0.4 N NaOH was added to the mixture and the resulting color was measured spectrophotometrically at 510 nm against a reagent blank. The intensity of the color reflects the concentration of the oxoacid present. A working standard of pyruvate was treated similarly and the activities of transaminases were calculated from the equation:

$$\mathrm{Enzyme}\;\mathrm{activity}\;\mathrm{(U}/\mathrm{L)}\;=\frac{\mathrm{Test}\;\mathrm{absorbance}}{\mathrm{Standard}\;\mathrm{absorbance}}\times \frac{\mathrm{Conc}.\;\mathrm{of}\;\mathrm{standard}}{\mathrm{Incubation}\;\mathrm{time}}\times \frac{1000}{\mathrm{Vol}.\;\mathrm{of}\;\mathrm{sample}}$$

#### Determination of plasma alkaline phosphatase

The plasma alkaline phosphatase (ALP) was analyzed according to the method of Kind and King 1954.[8] The assay principle is as follows: the substrate, disodium phenyl phosphate is hydrolyzed to phenol in the presence of the enzyme. The phenol condenses with aminoantipyrine then oxidized with potassium ferricyanide and the resulting red colored pigment monitored spectrophotometrically. Briefly, 1 mL sodium carbonate buffer, pH 10 was added to 1 mL, 0.01 M disodium phenyl phosphate substrate in a tube and the mixture was warmed at 37°C for 3 min. Plasma sample, 0.5 mL was added to the tube, mixed, and incubated at 37°C for 15 min, after which the reaction was stopped by adding 0.8 mL, 0.5 M NaOH solution. The hydrolytic product, phenol was condensed with 1.2 mL, 0.6% 4-aminoantipyrine, and then oxidized with 1 mL, 2.4% potassium ferricyanide to give to red colored complex, which was measured at 540 nm. The activity of alkaline phosphatase was calculated from the amount of phenol present. The same experiment was carried out for the standard in which 1 mL of 0.01 mg/mL phenol standard solution was substituted for the substrate.

#### Determination of total or direct bilirubin in plasma

Totalor direct bilirubin in plasma was determined using Jandrassik and Grof technique as described by Tolman *et al*., 1999.[9] For total bilirubin, a mixture of 0.2 mL plasma, 2 mL caffeine-benzoate reagent, and 0.5 mL freshly prepared diazotized sulfanilic acid reagent were placed in a tube, mixed, and left to stand for 10 min. Thereafter, 0.1 mL ascorbic acid, 1.5 mL alkaline tartrate, and 1 mL 0.05 mol/L hydrochloric acid solutions were added in succession and mixed. For the direct bilirubin level, the plasma was acidified with 1 mL, 0.05 mol/L hydrochloric acid and thereafter treated as in total bilirubin determination without caffeine-benzoate reagent. The absorbances of the blue-green solutions were measured against the reagent blank at 600 nm. The levels of total or direct bilirubin were determined from the calibration curve of bilirubin.

#### Total plasma protein

Protein concentration in the plasma was determined by biuret method.[10] Seven milliliters of reaction mixture contained 0.1 mL plasma sample, 2.9 mL deionized water, and 4.0 mL biuret reagent. For control, deionized water replaced the plasma sample. The standard mixture contained 0.25 mL 6% bovine serum albumin. The mixtures were left to stand at 25°C for 30 min and the absorbances were measured at 540 nm. An increasing intensity of violet color was related to higher percentage of protein concentration present in the reaction, calculated as

$$A_{T:\;30}/A_{S:\;30}\;\times \;\mathrm{conc}.\;\mathrm{of}\;S.$$

Where, A_T: 30_ is the absorbance of plasma sample, A_S: 30_ the absorbance of standard at 30 min reaction time, and S is the standard.

#### Plasma albumin determination

The plasma albumin was determined based on its reaction with bromocresol green.[11] Five milliliters bromocresol green buffered at pH 4.2 was added to 0.1 mL plasma properly mixed and incubated at 37°C for 10 min. The absorption of the dye–albumin complex was measured spectrophotometrically at 630 nm against a reagent blank.

#### Electrolyte determination

The plasma levels of sodium (Na^+^), potassium (K^+^), and chloride (Cl^–^) ions were determined using ion-selective electrode; in a Synchron CX_5_ automated system. The electrolyte reference for sodium ion consisted of sodium, 140 mmol/L, potassium, 4 mmol/L, chloride, 100 mmol/L, and carbon dioxide, 100 mmol/L. The carbon dioxide alkaline buffer for potassium ion consisted of potassium bicarbonate, 6 mmol / L and potassium chloride, 10 mmol/L. For chloride ion, the buffer reagent consisted of trisphosphate, 1.5 M. Calculation was made using the Nernst equation.[12]

#### Plasma bicarbonate determination

The pH electrode with a silicone rubber membrane was used to measure carbon dioxide by differential pH. The carbon dioxide acid reagent consisted of sulfuric acid, 0.6 M. The rate of change in the pH of the carbon dioxide acid reagent is directly proportional to the amount of carbon dioxide in the plasma.

#### Data and statistical analysis

Data are expressed as mean ± SEM (standard error of mean). Statistical analyses were performed using SPSS version 11 (SPSS Inc., 233 South Wacker Drive, Chicago, IL 60606-6412; Patent No. 7,023,453). Analyses of the data were performed using Student’s t test. Values were considered to differ significantly if the P value was less than 0.05 or 0.001.

## RESULTS

### Toxicity studies

**Figure 1:**
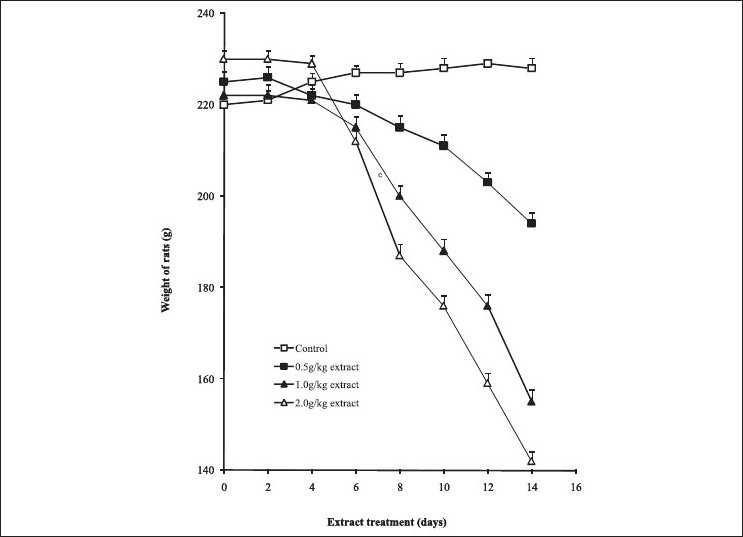
Weight loss pattern in rats at different days of treatments with the aqueous extract of *Vitex grandiflora*

The acute toxicity of the aqueous leaf extract of *V. grandifolia* in rats recorded no mortality even at a high dose of 2 g/kg body weight of the animal, thus LD_50_ could not be determined. However, daily administration of the aqueous extract of *V. grandifolia* to the rats for 14 days showed significant reduction in the body weight compared with the control (*P≤* 0.001, Figure 1). By visual observation, there was profuse increase in water intake, polyuria, puffiness of hair, and calmness in the test groups. The reduction in body weight was consistent with increase in doses of the extract.

### Histopathologic studies

**Figure 2:**
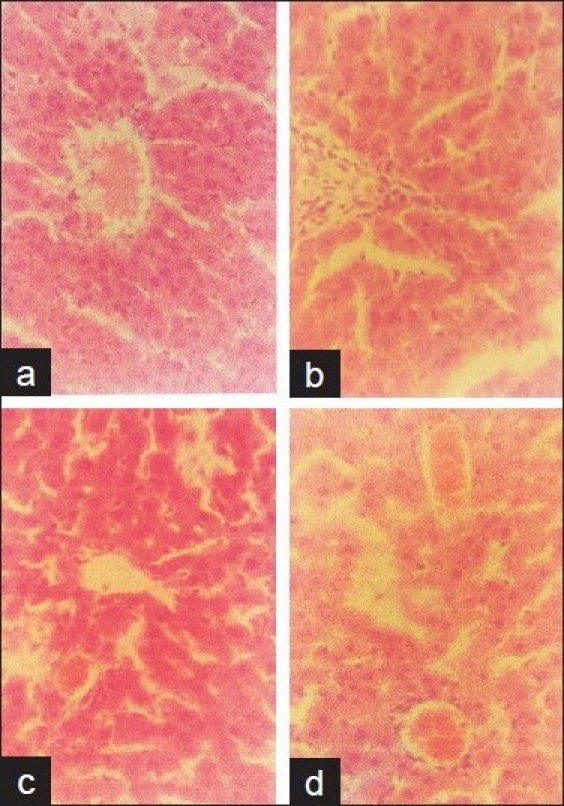
Histology of the liver after 14 days treatment with aqueous leaf extract of *Vitex grandifolia*. Control (a), 0.5 mg/kg b.wt. (b), 1 g/kg b.wt. (c), 2 g/kg b.wt. (d). Magnification ×400

**Figure 3:**
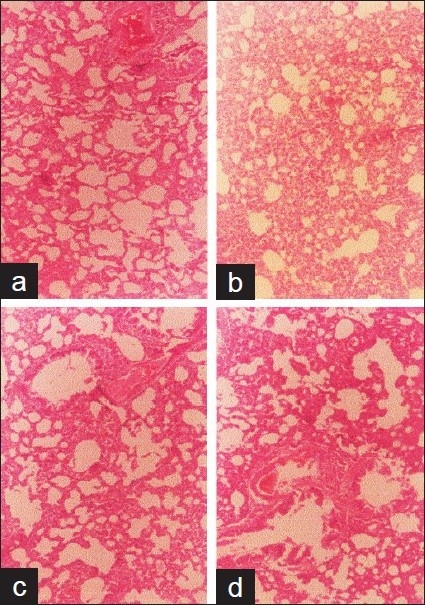
Histology of the lung after 14 days treatment with aqueous leaf extract of *Vitex grandifolia*. Control (a), 0.5 mg/kg b.wt. (b), 1 g/kg b.wt. (c), 2 g/kg b.wt. (d). Magnification ×100

**Figure 4:**
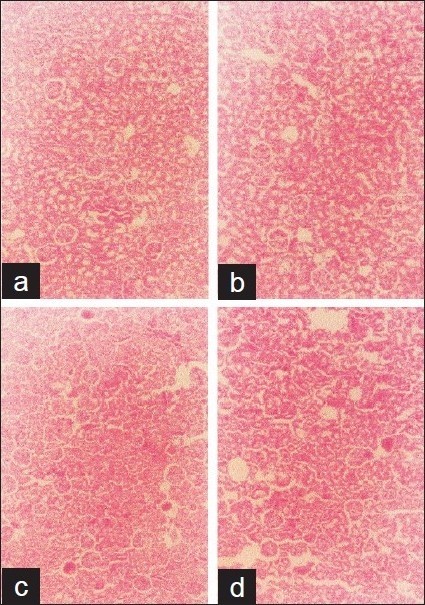
Histology of the kidney after 14 days treatment with aqueous leaf extract of *Vitex grandifolia*. Control (a), 0.5 mg/kg b.wt. (b), 1 g/kg b.wt. (c), 2 g/kg b.wt. (d). Magnification ×100

The results of the histologic study are shown in Figures 2–4. Except for the heart, there were significant alterations of the vital organs (liver, lung, and kidney) dose dependently after 14 days administration of the extract. The extract caused fatty liver, complete distortion of the liver architecture with portal infiltration of the inflammatory cells, and some areas of fibrosis, dilatation, and congestion of the portal vessel in some foci with edematous hepatocytes [Figure 2a–d]. In the lung, the treated groups showed severe pulmonary edema with rupture of the alveoli and infiltration of the mononuclear cells in the thin fibrous strands compared with the control [Figure 3a–d]. Severe edema, distortion of the architecture of the kidney with prominent vascular markings characterized the kidney of the rats treated with the extract [Figure 4a–d]. The severity of the effect on these organs increased with doses of the extract.

### Biochemical studies

Repeated intake of *V. grandifolia* significantly increased plasma electrolytes (Na^+^, K^+^, Cl^–^, HCO3^–^) dose dependently (*P* ≤ 0.001, Table 1). The liver enzyme activities are shown in table 2. There was a significant dose-dependent increase in the liver enzymes (ALT, AST, and ALP) in the test groups compared with the control (*P* ≤ 0.001). The results of this study showed that repeated administration of the aqueous extract of *V. grandifolia* presented a low level of direct bilirubin with an increase in the total bilirubin in the test groups compared with the control (*P*0.001, Table 2). The levels of albumin and protein in the test groups were also found to be lower than the control (*P* ≤ 0.001) after repeated administration of the aqueous extract of *V. grandifolia*. The mean plasma creatinine level in the test groups was significantly higher than the control (*P* ≤ 0.001).

**Table 1:** Changes in plasma electrolytes in rats after administration of the aqueous extract of *Vitex grandifolia*

Conc. (g/kg body weight)	Biochemical parameters (mmol/L)			
	Na^+^	K^+^	Cl^–^	HCO_3_^–^
Control	136. 13 ± 1.16^a^^b^	3.96 ± 1.06^b^	98.93 ± 2.24^b^	26.24 ± 1.45^a^^b^
0.50	148.34 ± 1.73^a^	5.61 ± 1.12^a^	121.11 ± 1.75	31.06 ± 1.56^a^
1.00	166.49 ± 1.24	6.47 ± 1.96^a^	147.58 ± 1.77^b^	55.11 ± 1.42
2.00	185.65 ± 1.68^b^	8.43 ± 2.03^b^	169.54 ± 1.82^b^	69.45 ± 1.47^b^

SEM, standard error of mean, Values represent mean ± SEM of 6 observations,

a *P* ≤0.05;

b *P* ≤ 0.001 (Student’s *t* test)

**Table 2:** Biochemical parameters in rats after oral administration of aqueous extract of *Vitex grandifolia*

Biochemical parameters	Control	Treated groups (dose in g/ kg body weight)		
		0.50	1.00	2.00
Total bilirubin (mmol/L)	10.33 ± 1.05^b^	25.07 ± 0.82	44.91 ± 1.07	71.68 ± 1.15^b^
Direct bilirubin (mmol/L)	2.15 ± 1.27^a^^b^	1.83 ± 1.21^a^	1.37 ± 1.51	0.96 ± 2.09^b^
Total proteins (g/L)	76.14 ± 2.18^b^	52.23 ± 1.75	31.16 ± 1.72	11.41 ± 1.83^b^
Albumin (g/L)	45.29 1.17^a^^b^	37.50 ± 1.57^a^	23.97 ± 1.26	15.70 ± 1.22^b^
Creatinine (mmol/L)	81.56 2.71	99.50 ± 2.23	112.85 ± 1.98	136.14 ± 2.13
ALP (U/L)	74.83 1.11^b^	88.18 ± 0.93	108.74 ± 1.09	129.36 ± 1.32^b^
AST (U/L)	25.61 2.21	39.11 ± 1.89^b^	44.74 ± 2.17^b^	72.71 ± 1.58
ALT (U/L)	21.53 ± 2.21^b^	41.73 ± 1.85^b^	58.44 ± 2.16^b^	68.27 ± 2.27^b^

ALP, alkaline phosphatase; AST, aspartate transaminase; ALT, alanine transaminase; SEM, standard error of mean, Values represent mean ± SEM of 6 observations

a *P* ≤0.05;

b *P* ≤ 0.001 (Student’fs *t* test)

## DISCUSSION

The reduction in body weight of the treated animal, which was consistent with increase in doses of the extract may be due to increased dehydration deduced by excessive wetting and polyuria. Electrolytes play an important role in maintaining homeostasis within the body. They help to regulate proper heart, muscle, and neurologic function, fluid balance, oxygen delivery, and acid–base balance.[13] Excessive fluid loss in the treated group may be due to increase in plasma Na^+^. The high mean plasma concentration of Na^+^ causes hypertonicity of the blood, which results in a shift of water out of the cells making the cells dehydrated. Typical observed effect of high Na^+^ was increased water intake. The mechanism by which K^+^ concentration is increased in this study is unclear. However, it may give an insight into its use as a potassium sparing diuretic. The fact that *V. grandifolia* is used locally as an antidiabetic (Dosa, 2007, personal communication) may explain the high K^+^ level in the serum. Insulin is a stimulator of cellular K^+^ uptake,[14,15] therefore a fall in glucose would decrease plasma insulin, which would in turn reduce cellular K^+^ uptake, thus increasing plasma K^+^ level. Chloride (Cl^–^) and bicarbonate ions (HCO3^–^) are negative electrolytes that regulate blood pressure and blood pH levels, respectively. Their increase was consistent with the increase in Na^+^ and K^+^ in the serum.

The biochemical parameters were used as indices for assessing organ dysfunction or damage as could arise in toxicity. The liver plays an important role in many metabolic processes; any disturbance in the liver would affect the normal level of measurable biochemical parameters in this organ. AST, ALT, and ALP are marker enzymes present in high concentrations in the liver, when liver cells are inflamed or damaged, these enzymes leak into the blood stream leading to a rise in the plasma level of these enzymes.[16,17] ALT is selectively a liver parenchymal enzyme than AST and a sensitive indicator of acute liver damage.[18] Thus elevation of these enzymes in our study indicates inflammation or damage of the liver cells. Nerbert[19] reported that damaged liver cells lose the ability to conjugate bilirubin or remove unconjugated bilirubin from the blood thus an increase in unconjugated bilirubin in the serum. The fact that the direct bilirubin is low with resulting increase in total bilirubin in this study indicates liver damage. The low levels of albumin or protein in the plasma may also explain damage to the liver and diminished synthetic function of the liver. These results taken together strongly indicate liver damage in the test groups compared with the control.

There were significant alterations in the architecture of the vital organs (liver, lung, and kidney). The observed alterations in these organs may be due to damage to these organs by the aqueous extract of *V. grandifolia*. These results taken altogether would give an insight into the toxic nature of the aqueous leaf extract of *V. grandifolia* on long-term administration despite its claimed usefulness in folklore medicine.
